# Circulating tumor cells as early predictors of metastatic spread in breast cancer patients with limited metastatic dissemination

**DOI:** 10.1186/s13058-014-0440-8

**Published:** 2014-09-16

**Authors:** Mario Giuliano, Antonio Giordano, Summer Jackson, Ugo De Giorgi, Michal Mego, Evan N Cohen, Hui Gao, Simone Anfossi, Beverly C Handy, Naoto T Ueno, Ricardo H Alvarez, Sabino De Placido, Vicente Valero, Gabriel N Hortobagyi, James M Reuben, Massimo Cristofanilli

**Affiliations:** 1 Department of Clinical Medicine and Surgery, University Federico II, Naples, Italy; 2 Lester and Sue Smith Breast Center, Baylor College of Medicine, Houston, TX USA; 3 Department of Hematopathology, The University of Texas MD Anderson Cancer Center, Houston, TX USA; 4 Department of Internal Medicine, Medical University of South Carolina, Charleston, SC USA; 5 Breast Medical Oncology, The University of Texas MD Anderson Cancer Center, Houston, TX USA; 6 Medical Oncology, Istituto Scientifico Romagnolo per lo Studio e la Cura dei Tumori, Meldola, FC Italy; 7 Department of Medical Oncology, School of Medicine, Comenius University, Bratislava, Slovakia; 8 Laboratory Medicine, The University of Texas MD Anderson Cancer Center, Houston, TX USA; 9 Breast Program, Sidney Kimmel Cancer Center, Thomas Jefferson University, 1025 Walnut Street, Suite 700, Philadelphia, 19107 PA USA

## Abstract

**Introduction:**

Traditional factors currently used for prognostic stratification do not always adequately predict treatment response and disease evolution in advanced breast cancer patients. Therefore, the use of blood-based markers, such as circulating tumor cells (CTCs), represents a promising complementary strategy for disease monitoring. In this retrospective study, we explored the role of CTC counts as predictors of disease evolution in breast cancer patients with limited metastatic dissemination.

**Methods:**

A total of 492 advanced breast cancer patients who had a CTC count assessed by CellSearch prior to starting a new line of systemic therapy were eligible for this analysis. Using the threshold of 5 CTCs/7.5 ml of blood, pretreatment CTC counts were correlated in the overall population with metastatic site distribution, evaluated at baseline and at the time of treatment failure, using Fisher’s exact test. Time to visceral progression and time to the development of new metastatic lesions and sites were estimated in patients with nonvisceral metastases and with single-site metastatic disease, respectively, by the Kaplan-Meier method. Survival times were compared between groups according to pretreatment CTC count by logrank test.

**Results:**

In the overall population, a pretreatment level ≥5 CTCs/7.5 ml was associated with an increased baseline number of metastatic sites compared with <5 CTCs/7.5 ml (*P* = 0.0077). At the time of treatment failure, patients with ≥5 CTCs/7.5 ml more frequently developed new metastatic lesions and sites compared with those with <5 CTCs/7.5 ml (development of new lesions: *P* = 0.0002; development of new sites: *P* = 0.0031). Among patients with disease originally confined to nonvisceral sites, ≥5 CTCs/7.5 ml was associated with remarkably shorter time to visceral metastases (*P* = 0.0021) and overall survival (*P* = 0.0006) compared with <5 CTCs/7.5 ml. In patients with single-site metastatic disease, ≥5 CTCs/7.5 ml was associated with a significant reduction of the time to development of new metastatic sites (*P* = 0.0051) and new lesions (*P* = 0.0002) and with worse overall survival (*P* = 0.0101).

**Conclusion:**

Our results suggest that baseline CTC counts can be used as an early predictor of metastatic potential in breast cancer patients with limited metastatic dissemination.

**Electronic supplementary material:**

The online version of this article (doi:10.1186/s13058-014-0440-8) contains supplementary material, which is available to authorized users.

## Introduction

Breast cancer mortality has decreased considerably over the past two decades as a result of earlier diagnosis and major treatment advances in the adjuvant and metastatic settings [1]. Despite this progress, metastatic disease is still largely considered an incurable condition, and 5-year survival rates are <25% [1]. However, metastatic breast cancer is a heterogeneous disease, and long-term patient outcomes can be influenced by various biological features, as well as by the extent and site of metastatic disease. On the one hand, widespread visceral disease is typically associated with symptoms leading to deterioration of performance status and short survival [2]. On the other hand, limited metastatic dissemination, primarily confined to nonvisceral tissues, is more frequently associated with an indolent disease course and prolonged survival [3]-[5]. Nevertheless, the current standard assessment of metastatic disease by morphological and functional imaging does not provide adequate information on tumor biology and the presence of micrometastases, limiting the possibility to predict tumor metastatic potential [3].

During the past decade, several techniques capable of detecting and quantifying circulating tumor cells (CTCs) in cancer patients have been developed [4],[5]. It has been proposed that subpopulations of CTCs with tumor-initiating potential act as a central mediator of metastatic dissemination, giving rise to the formation of distant micrometastases, which subsequently generate overt detectable and frequently measurable lesions [6]. In support of this theory, multiple studies have shown that ≥5 CTCs/7.5 ml of blood, counted using the CellSearch System (Janssen Diagnostics, Raritan, NJ, USA) and evaluated before starting systemic treatment, is associated with poor outcome in patients with metastatic breast cancer [7]-[10]. In addition, high CTC counts are associated with greater metastatic tumor burden, expressed as the number of metastatic sites [8],[11]. Importantly, despite this association, the prognostic value of CTCs is independent from the initial number of metastatic sites [8],[11]. This may suggest that the negative prognostic impact of high CTC counts is not merely expression of the overt tumor burden, but also may reflect higher biological aggressiveness and presence of undetectable micrometastatic disease, and ultimately may predict a greater tendency to metastatic spread.

Therefore, we hypothesized that CTC counts, when evaluated prior to starting systemic treatment, represent an early marker of metastatic spread and are useful primarily in patients with limited metastatic dissemination and potentially eligible for locoregional treatments with a curative intent [12]-[14]. To test our hypothesis, we analyzed the patterns of recurring metastatic dissemination in patients with advanced breast cancer who had a CTC count before starting a new line of systemic treatment.

## Methods

### Study design

In this study, we conducted a retrospective analysis of a preexisting database including 517 metastatic breast cancer patients treated at The University of Texas MD Anderson Cancer Center between September 2002 and November 2009. All the patients had a CTC count using the CellSearch System before starting a new line of systemic treatment. From among the overall population, 492 women (95%) were selected for this analysis because they had documented radiological follow-up. Eligible patients were categorized into two groups according to baseline CTC counts using the well-established threshold of 5 CTCs/7.5 ml of blood [7],[15] (<5 CTCs/7.5 ml versus ≥5 CTCs/7.5 ml of blood). Radiologic disease assessments were performed in line with institutional guidelines, and progression of disease (PD) was defined according to the Response Evaluation Criteria In Solid Tumors (RECIST) [16]. Reports based on radiologic assessments performed in each patient after baseline CTC evaluation were reviewed. The type of PD was recorded and classified as appearance of new metastatic lesions, either within the preexisting sites or in new metastatic sites, or as dimensional increase of the preexisting lesions. These analyses were performed in the overall population and selectively in patients with limited metastatic dissemination at baseline, defined as disease confined to nonvisceral organs or to a single organ. The Institutional Review Board at MD Anderson Cancer Center approved the study (DR10-0227) and granted a waiver of informed consent, considering the retrospective nature of this analysis. Clinical data from the MD Anderson Cancer Center's electronic medical records were collected by two physicians (AG and MG).

### Isolation and enumeration of circulating tumor cells

Peripheral blood samples (7.5 ml) collected within 30 days before starting any systemic treatment were drawn into CellSave tubes and processed within 72 hours of collection. The standardized US Food and Drug Administration-cleared CellSearch System was used to isolate and count CTCs as previously reported [17]. CTCs were defined as nucleated, epithelial cell adhesion molecule (EpCAM)-positive cells, expressing cytoplasmic cytokeratins 8, 18 and 19, but lacking expression of the common leukocyte antigen CD45. All CTC evaluations were performed by qualified personnel in a pathology laboratory certified in accordance with the Clinical Laboratory Improvement Amendments.

### Statistical analysis

The rate of new metastatic sites and new metastatic lesions, the number of new metastatic sites developed at the time of first PD (one or at least two) and the differences in the organ distribution of metastatic sites were compared between the two CTC groups using Fisher’s exact test. The number of metastatic sites present at baseline (one, two or three or more) and the type of systemic treatment were compared between the CTC groups using Pearson’s χ^2^ test. Time to PD was defined as the interval between baseline CTC count and PD or death. Overall survival was defined as the time elapsed between initial CTC assessment and patient death. In patients with disease confined to a single organ, time to new metastatic sites was calculated from baseline to the appearance of new metastatic sites. In the same group, time to new metastatic lesions indicated the interval between baseline CTC count and development of new metastatic lesions, either in preexisting sites or at new sites. Time to visceral disease was defined as the interval between baseline and development of visceral metastases in patients with disease initially confined to nonvisceral organs. For the estimation of all of the aforementioned survival times, in the absence of the specific event, patients were censored at the date of the last follow-up. Each survival time was estimated using the Kaplan-Meier product limit method and compared between the CTC groups (<5 CTCs/7.5 ml versus ≥5 CTCs/7.5 ml) by logrank test. Multivariate survival analysis was performed using Cox’s regression. All the statistical analyses, performed using the PASW Statistical Analysis for Social Sciences (SPSS) statistics software (SPSS, Chicago, IL, USA), were two-sided, and *P*-values <0.05 were considered statistically significant.

## Results

### Pretreatment circulating tumor cell counts correlated with extent and site of metastatic disease at baseline

Baseline patient and tumor characteristics stratified by CTC counts are reported in Table 1. Among the patient sample, 303 (61.6%) had <5 CTCs/7.5 ml and 189 (38.4%) had ≥5 CTCs/7.5 ml. No significant difference in terms of treatment strategy (systemic treatment only versus additional locoregional treatment) and line of systemic treatment was found between the CTC groups (Table 1). Conversely, the type of systemic treatment significantly differed, with a higher percentage of patients receiving chemotherapy and a lower percentage receiving endocrine and other therapies in the group with ≥5 CTCs/7.5 ml compared with <5 CTCs/7.5 ml (*P* = 0.0041) (Table 1). The distribution of the immunohistochemically defined tumor subtypes stratified by CTC value was previously reported and did not show significant differences between the CTC groups [8]. Also, in line with previously published reports [8],[18], high baseline CTC counts were associated with greater metastatic tumor burden and higher frequency of bone involvement (Table 1). The percentage of cases with disease confined to lymph nodes and/or soft tissues was significantly higher in the group with low CTCs compared with that of patients with high CTCs (Table 1).

**Table 1:** **Baseline patient and tumor characteristics stratified by circulating tumor cell count**

Variable	Overall,*N*(%)	CTCs <5/7.5 ml,*n*(%)	CTCs ≥5/7.5 ml,*n*(%)	*P*-value
Overall population	492 (100)	303 (61.6)	189 (38.4)	-
Treatment strategy				
Systemic treatment only	467 (94.9)	286 (94.7)	180 (95.2)	ns
Additional locoregional treatment	25 (5.1)^a^	16 (5.3)	9 (4.8)	
Line of treatment				
First	232 (47.2)	139 (45.9)	93 (49.2)	ns
Second or later	260 (52.8)	164 (54.1)	96 (50.8)	
Type of systemic treatment				
Chemotherapy	376 (76.4)	218 (71.9)	158 (83.6)	0.0041
Endocrine therapy	103 (20.9)	73 (24.1)	30 (15.9)	
Other	13 (2.7)	12 (4.0)	1 (0.5)	
Number of metastatic sites				
1	146 (29.7)	104 (34.3)	42 (22.2)	0.0077
2	145 (29.5)	89 (29.4)	56 (29.6)	
≥3	201 (40.8)	110 (36.3)	91 (48.2)	
Distribution of metastatic sites				
Lymph nodes/soft tissues^b^	58 (11.8)	48 (15.8)	10 (5.3)	0.0003
Bone^c^	326 (66.2)	172 (56.8)	154 (81.5)	<0.0001
Viscera^d^	306 (62.2)	184 (60.7)	122 (64.6)	ns
Brain^e^	37 (7.5)	23 (7.6)	14 (7.4)	ns

^a^Additional locoregional treatments: surgery 15 (3.0%); radiation therapy 8 (1.6%); other 2 (0.4%). ^b^Lymph nodes/soft tissue only. ^c^Bone with or without other sites. ^d^Visceral organs (including brain) with or without other sites. ^e^Brain with or without other sites. CTCs, Circulating tumor cells; ns, Statistically nonsignificant.

### Pretreatment circulating tumor cell counts correlated with magnitude of metastatic dissemination at the time of disease progression

**Figure 1:**
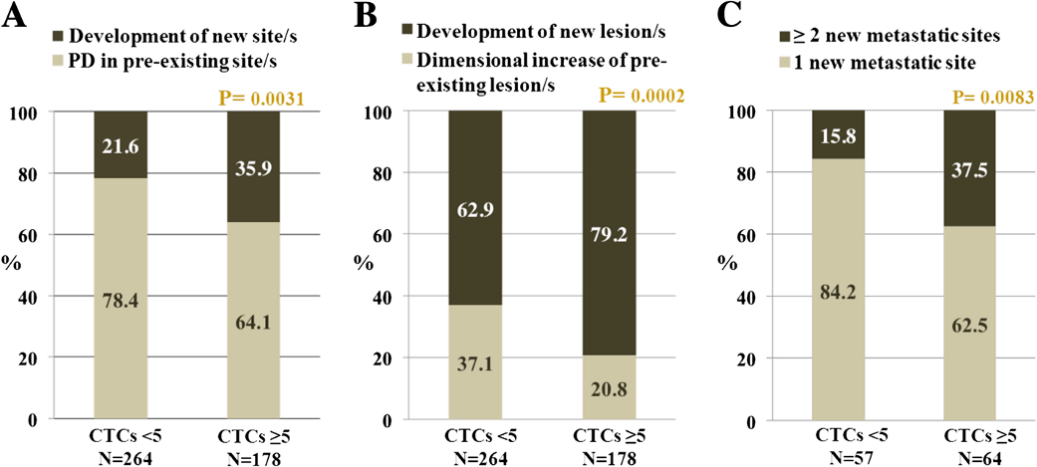
**Association of baseline circulating tumor cell counts with metastatic spread in the overall population. (A)** and **(B)** Rates of development of new metastatic sites **(A)** and new lesions **(B)** refer to the first progression of disease (PD) after baseline in the overall population (*N* = 492), stratified by circulating tumor cell (CTC) count. **(C)** Number of new metastatic sites that had developed by the first PD after baseline in patients whose disease had progressed in new sites (*n* = 121), stratified by CTC count.

At the time of this analysis, the median follow-up among living patients was 32.2 months in the overall population (33.4 months in the group with <5 CTCs/7.5 ml and 30.6 months in the group with ≥5 CTCs/7.5 ml). Among the two groups, 264 patients (87.1%) with <5 CTCs/7.5 ml and 178 (94.2%) with ≥5 CTCs/7.5 ml had experienced PD before the last follow-up visit. Median times to the first PD were 6.8 months (95% CI: 5.8 to 7.8 months) in subjects with <5 CTCs/7.5 ml and 5.9 (95% CI: 5.0 to 6.8 months) in those with ≥5 CTCs/7.5 ml (*P* = 0.0059). The extent of metastatic spread occurring at the time of the first PD after CTC assessment was evaluated and correlated with the baseline CTC counts. The development of new metastatic sites was significantly more frequent among patients with ≥5 CTCs/7.5 ml compared to those with <5 CTCs/7.5 ml (*P* = 0.0031) (Figure 1A). Similarly, the development of new metastatic lesions, either within the preexisting sites or in new metastatic sites, was more frequent in the group of patients with ≥5 CTCs/7.5 ml (*P* = 0.0002) (Figure 1B). In addition, the number of new metastatic sites that developed at the time of first PD was significantly higher in the patients with ≥5 CTCs/7.5 ml compared to those with <5 CTCs/7.5 ml (*P* = 0.0083) (Figure 1C). All of these findings translated into a marked difference in long-term outcomes, with median overall survival of 31.5 months (95% CI: 23.9 to 39.1 months) for patients with <5 CTCs/7.5 ml versus 19.1 (95% CI: 15.9 to 22.3 months) for those with ≥5 CTCs/7.5 ml (*P* = 0.0001).

### Pretreatment circulating tumor cell counts correlated with visceral disease spread at the time of disease progression

At the time of the first PD, similar to baseline, the frequency of cases with metastatic disease confined to lymph nodes and/or soft tissues was higher among patients with <5 CTCs/7.5 ml than in patients with ≥5 CTCs/7.5 ml (*P* = 0.0009) (Table 2). In addition, after PD, the rate of visceral metastases became significantly higher in the group of patients with ≥5 CTCs/7.5 ml (*P* = 0.0114) (Table 2). Of note, we also observed a trend for a higher frequency of brain metastases in patients with ≥5 CTCs/7.5 ml (*P* = 0.0865) (Table 2).

**Table 2:** **Distribution of metastatic sites after treatment failure stratified by pretreatment circulating tumor cell count** ^**a**^

	Overall,*N*(%)	CTCs <5/7.5 ml,*n*(%)	CTCs ≥5/7.5 ml,*n*(%)	*P*-value
Lymph nodes/soft tissues^b^	48 (9.8)	40 (13.2)	8 (4.2)	0.0009
Bone^c^	337 (68.5)	180 (59.4)	157 (83.1)	<0.0001
Viscera^d^	344 (69.9)	199 (65.7)	145 (76.7)	0.0114
Brain^e^	59 (12.0)	30 (9.9)	29 (15.3)	0.0865

^a^Treatment failure was defined as first disease progression after the baseline CTC evaluation. ^b^Lymph nodes/chest soft tissue only. ^c^Bone with or without other sites. ^d^Visceral organ (including brain) with or without other sites. ^e^Brain with or without other sites.

**Figure 2:**
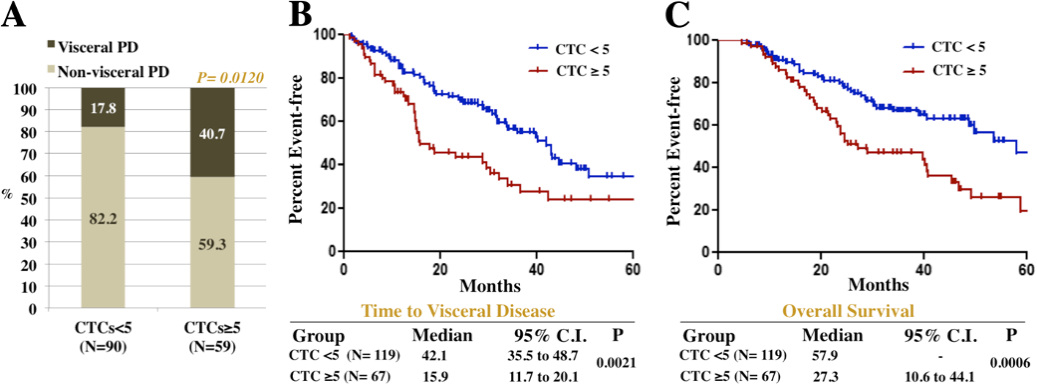
**Association of baseline circulating tumor cell counts with visceral disease spread. (A)** Types of the first progression of disease (PD) occurring after baseline in 149 patients with disease initially confined to nonvisceral organs, stratified by circulating tumor cell (CTC) count. Thirty-seven (19.9%) of the total of one hundred eighty-six patients without baseline visceral metastases had not developed PD before the last follow-up visit. **(B)** and **(C)** Time to visceral disease **(B)** and overall survival **(C)** in the 186 patients without visceral metastases, stratified by CTC count.

To further assess the correlation between CTC counts and visceral disease spread, we analyzed the patterns of metastatic dissemination selectively in 186 patients (37.8%) with disease initially confined to nonvisceral organs. Of those, 119 (64%) had <5 CTCs/7.5 ml and 67 (36%) had ≥5 CTCs/7.5 ml. A total of 149 patients with nonvisceral metastases experienced PD by the time of their last visit. Among these patients, the development of visceral metastases was significantly more frequent in the group with ≥5 CTCs/7.5 ml compared to the group with <5 CTCs/7.5 ml (40.7% versus 17.8%, respectively; (*P* = 0.012) (Figure 2A). Also, the time to the development of visceral disease was remarkably different between the CTC groups (42.1 months versus 15.9 months in women with <5 CTCs/7.5 ml versus ≥5 CTCs/7.5 ml, respectively; *P* = 0.0021) (Figure 2B). The multivariate analysis demonstrated that the effect of CTC counts on time to visceral disease was independent from other prognostic variables, including estrogen receptor (ER) and human epidermal growth factor receptor 2 (HER2) status, baseline number of metastatic sites and presence of bone metastases (Additional file 1: Table S1). By the time of the last follow-up visit, 53 (44.5%) of the 119 patients with <5 CTCs/7.5 ml and only 14 (20.9%) of the 67 with ≥5 CTCs/7.5 ml were free of visceral disease (*P* = 0.0229). As expected, all of these findings were accompanied by a highly significant difference in overall survival between the CTC groups (57.9 months for patients with CTCs <5 CTCs/7.5 ml versus 27.3 months for those with ≥5 CTCs/7.5 ml; *P* = 0.0006).

### Pretreatment circulating tumor cell counts correlated with metastatic dissemination in patients with disease originally confined to a single organ

**Figure 3:**
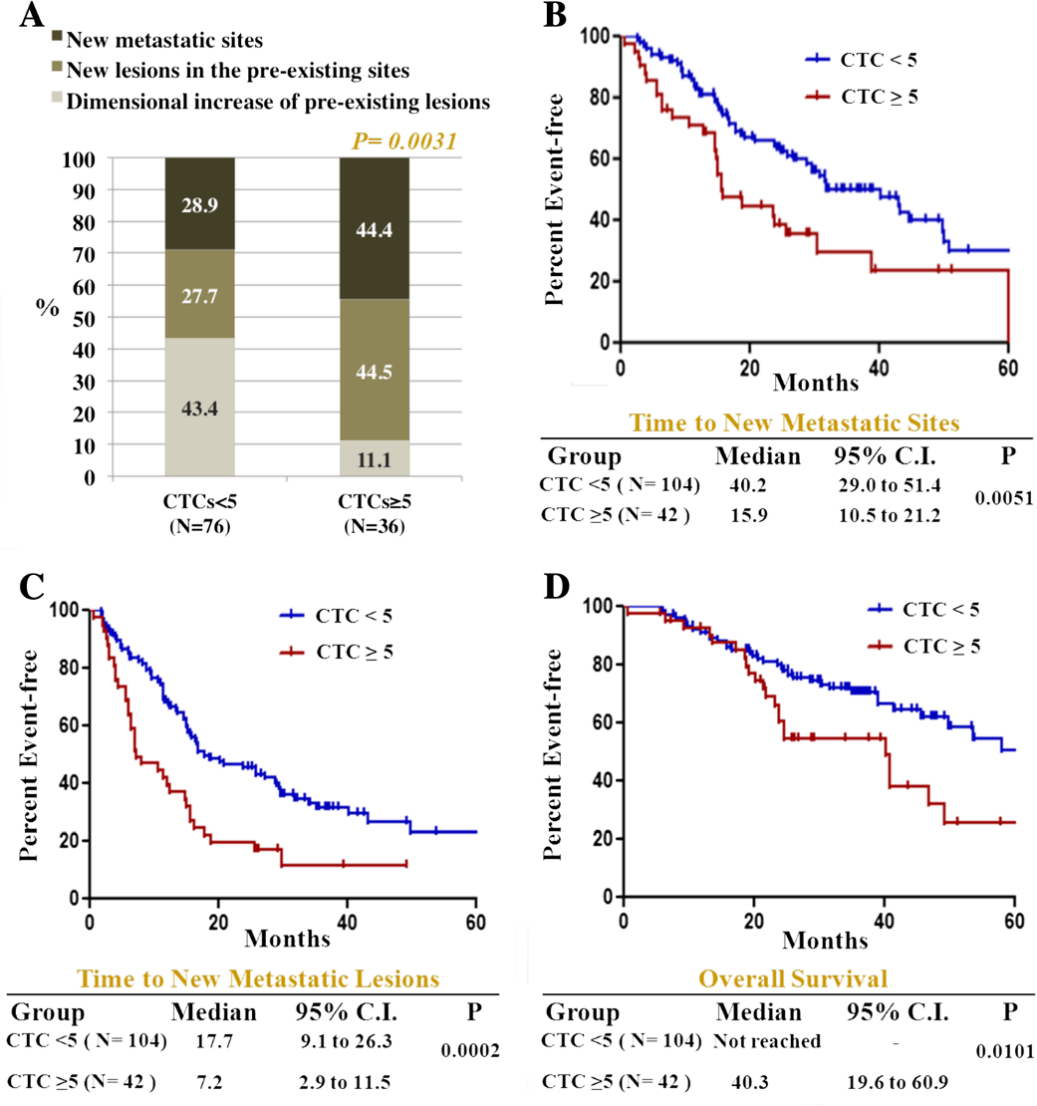
**Association of baseline circulating tumor cell counts with metastatic spread in patients with single-site disease. (A)** Type of the first progression of disease (PD) occurring after baseline in 112 patients with single metastatic site disease, stratified by circulating tumor cell (CTC) count. Thirty-four (23.3%) of the total one hundred forty-six patients with single-site disease had not developed PD before the last follow-up visit. **(B)**, **(C)** and **(D)** Time to new metastatic sites **(B)**, time to new metastatic lesions **(C)** and overall survival **(D)** in the 146 patients with single metastatic site disease, stratified by CTC count.

The impact of high CTC counts on metastatic dissemination was also evaluated selectively in 146 patients (29.7%) with disease originally confined to a single organ. In this group, 104 women (71.2%) had <5 CTCs/7.5 ml and 42 (28.8%) had ≥5 CTCs/7.5 ml at the baseline evaluation (Table 3). No statistically significant difference was found between the two CTC groups in terms of treatment strategy (systemic treatment alone or systemic treatment plus additional locoregional treatment) and type of systemic treatment (chemotherapy, endocrine therapy or other). On the contrary, the anatomical distribution of the metastatic sites was significantly different between the CTC groups, with bone metastases more frequent in patients with ≥5 CTCs/7.5 ml and soft-tissue/lymph node involvement more frequent among those with <5 CTCs/7.5 ml (*P =* 0.023) (Table 3). Median time to first PD was 12.2 months for patients with <5 CTCs/7.5 ml and 7.1 months for those with ≥5 CTCs/7.5 ml (*P* = 0.0373). Among the patients who experienced PD, those with ≥5 CTCs/7.5 ml more frequently developed new metastatic lesions, either within the preexisting sites or in new metastatic sites, compared to patients with <5 CTCs/7.5 ml (88.9% versus 56.6%, respectively; *P* = 0.0031) (Figure 3A). Moreover, the time to the development of new metastatic sites was remarkably longer in patients with low CTC counts compared to those with high counts (40.2 months versus 15.9 months for patients with <5 CTCs/7.5 ml versus ≥5 CTCs/7.5 ml, respectively; *P* = 0.0051) (Figure 3B). Similarly, there was a striking difference in the length of time to development of new metastatic lesions between the two CTC groups (17.7 months for patients with <5 CTCs/7.5 ml versus 7.2 months for those with ≥5 CTCs/7.5 ml; *P* = 0.0002) (Figure 3C). Importantly, the multivariate analysis showed that these differences were not dependent on other variables, including ER and HER2 status and presence of visceral or bone metastases at baseline (Additional file 1: Table S1). Unsurprisingly, all of these findings translated to a significant difference in long-term outcomes (overall survival: 40.3 months versus >60 months for patients with ≥5 CTCs/7.5 ml versus <5 CTCs/7.5 ml, respectively; *P* = 0.0101) (Figure 3D).

**Table 3:** **Baseline tumor features and treatments in patients with disease confined to a single organ** ^**a**^

Variable	Overall,*N*(%)	CTCs <5/7.5 ml,*n*(%)	CTCs ≥5/7.5 ml,*n*(%)	***P***-value
Overall population	146 (100)	104 (71.2)	42 (28.8)	-
HR status				
Positive	99 (67.8)	67 (64.4)	32 (76.2)	ns
Negative	47 (32.2)	37(35.6)	10 (23.8)	
HER2 status				
HER2 amplified/overexpressed	30 (20.6)	22 (21.2)	8 (19.1)	ns
HER2 normal	116 (79.4)	82 (78.8)	34 (80.9)	
Treatment strategy				
Systemic treatment only	129 (88.4)	91 (87.5)	38 (90.5)	ns
Additional locoregional treatment	17 (11.6)^b^	13 (12.5)	4 (9.5)	
Type of systemic treatment				
Chemotherapy	101 (69.2)	70 (67.3)	31 (73.8)	ns
Endocrine therapy	41 (28.1)	31 (29.8)	10 (23.8)	
Other	4 (2.7)	3 (2.9)	1 (2.4)	
Distribution of metastatic sites				
Lymph nodes/soft tissues	50 (34.2)	41 (39.4)	9 (21.4)	0.023
Bone	68 (46.6)	41 (39.4)	27 (64.3)	
Viscera	28 (19.2)	22 (21.2)	6 (14.3)	

^a^CTCs, Circulating tumor cells; HER2, Human epidermal growth factor receptor 2; HR, Hormone receptor; ns, Statistically nonsignificant. ^b^Additional locoregional treatments: surgery in 12 patients (8.2%), radiation therapy in 4 patients (2.7%) and other for 1 patient (0.7%).

## Discussion

Systemic metastatic spread represents the main cause of breast cancer-related morbidity and death. Thus, for patients in the advanced stages of disease, it is critical to improve treatment strategies that may affect not only proliferation but also migration and invasion, important features of the metastatic process. The traditional factors currently used for prognostic stratification and treatment decisions in the advanced stages of breast cancer, including hormone receptor and HER2 status, site and extent of metastatic burden, and length of disease-free interval, often do not adequately predict treatment response and disease evolution. In addition, treatment aggressiveness is still debated in the presence of disease confined to nonvisceral organs or asymptomatic visceral metastases. In this scenario, the use of blood-based disease monitoring, such as CTC assessment, may represent a complementary and informative strategy [19]-[23]. Recently, the results of a prospective clinical trial confirmed the strong prognostic value of CTCs in metastatic breast cancer patients [10],[24]. Moreover, a large pooled analysis of individual patient data showed that adding CTC count status to clinicopathological predictive models significantly improved survival prognostication in advanced breast cancer [24]. The results of our present study suggest a novel role for CTCs as predictors of disease evolution in patients with advanced breast cancer, particularly those with limited metastatic dissemination. We found that pretreatment CTC counts ≥5/7.5 ml correlated with greater metastatic dissemination at the time of treatment failure, owing to more frequent appearance of new metastatic lesions and sites. Also, the extent of the newly developed metastatic burden was greater in patients with ≥5 CTCs/7.5 ml compared with those with <5 CTCs/7.5 ml [6]. We can hypothesize that elevated CTC numbers, when observed prior to starting systemic treatment, may reveal greater propensity to metastatic seeding and more extensive micrometastatic disease and thus may function as an early predictor of overt metastatic spread. This further emphasizes the need to investigate the molecular features of these cells in addition to their prognostic value. Expectedly, CTC counts ≥5/7.5 ml were also associated with lower frequency of disease confined to nonhematogenous metastatic sites, such as lymph nodes and soft tissues. Importantly, among the patients with nonvisceral metastases in our sample, ≥5 CTCs/7.5 ml predicted higher risk of visceral progression. This finding could have important clinical implications. Indeed, it is generally recommended that aggressive treatment strategies, such as chemotherapy-based regimens, are needed in cases of widespread visceral metastases and rapidly progressive disease [25],[26]. On the basis of our results, it can be proposed that pretreatment CTC counts may identify in advance patients who have higher risk of developing widespread visceral disease and consequently might benefit from early administration of more aggressive systemic treatments. Nevertheless, the retrospective nature of our analysis suggests the need to prospectively validate the use of CTCs as surrogate markers of metastatic potential, together with the investigation of new therapeutic strategies aimed at targeting biological properties of CTCs, with the goal of preventing or delaying PD and possibly improving patient outcomes [27],[28].

The link between micrometastatic disease and CTCs, as well as their capability to predict overt metastatic dissemination, may have important implications in the clinical management of patients with single-site metastatic disease. Indeed, among patients with this specific condition, pretreatment CTC counts ≥5/7.5 ml were correlated with higher risk of developing new metastatic sites and lesions at the time of treatment failure. This may imply limited value of combined therapeutic modalities that include local therapies in patients with high CTC counts. However, 43% of patients with single-site disease and <5 CTCs/7.5 ml experienced dimensional increases in the preexisting lesions when treatment resistance occurred. Hence, pretreatment CTC counts <5/7.5 ml may identify a subgroup of patients with single-organ disease who have higher probability of maintaining this condition for a longer time upon systemic treatment and for whom locoregional ablative procedures, if feasible, can contribute to symptom control and prolong survival [12]-[14].

The retrospective nature of the analyses and the lack of CTC molecular profiling represent the main limitations of this study, principally because molecular heterogeneity of CTCs may influence their role in metastatic seeding. In particular, specific subpopulations of CTCs undergoing molecular reprogramming known as the epithelial-to-mesenchymal transition (EMT) [29],[30] process lose their epithelial differentiation and acquire a mesenchymal phenotype, with increased invasion capabilities and stemness properties [30],[31]. The results of several studies suggest that CTCs with EMT features are the major effectors of metastatic seeding and are responsible for tumor progression [32]-[35]. Importantly, in the presence of the EMT process and loss of epithelial markers, EpCAM-based isolation methods, such as the CellSearch System, do not perform adequately. Thus, sensitive new strategies for CTC isolation and analysis are needed in order to thoroughly use these cells for prediction of disease evolution [36],[37].

Despite these limitations, our study is clinically relevant because we propose, for the first time to our knowledge, a potential marker of disease spread in breast cancer patients with limited metastatic dissemination.

## Conclusion

Our findings suggest a new potential role for pretreatment CTC counts as early predictors of metastatic potential. This type of evaluation may be useful for improved risk stratification in patients with nonvisceral disease and single-organ metastatic involvement. Considering the limited number of patients evaluated in our study, our results warrant larger studies based on CTC enumeration and molecular profiling to develop novel treatment decision tools for breast cancer patients with limited metastatic dissemination.

## Authors' contributions

MG and AG conceived of and designed the study, collected clinical data, performed data analysis and interpretation, and drafted the manuscript. SJ performed the CellSearch CTC analysis of all patients and contributed to data interpretation and manuscript drafting and revision. UDG and MM collected clinical data and contributed to data interpretation and manuscript drafting and revision. EC and HG performed data analysis and contributed to data interpretation and manuscript drafting and revision. SA performed data analysis and contributed to manuscript drafting and revision. BCH performed pathological review of CTC images in the Clinical Laboratory Improvement Amendments-certified pathology laboratory and contributed to data interpretation and manuscript revision. NTU, RHA, VV and GNH provided clinical assessment of patients and contributed to analysis, study conception and manuscript revision. SDP performed data analysis and contributed to analysis, study conception and manuscript revision. JMR and MC conceived of and designed the study, performed data analysis and interpretation, and contributed to manuscript drafting and revision. All authors substantially participated in manuscript revision. All authors agree to be accountable for all aspects of the work in ensuring that questions related to the accuracy or integrity of any part of the work are appropriately investigated and resolved. All authors read and approved the final manuscript.

## Authors' information

The authors’ affiliations at the time of study design, data collection and analysis were as follows. MG, UDG, MM and ENC: Department of Hematopathology, The University of Texas MD Anderson Cancer Center, Houston, TX, USA. MC: Breast Medical Oncology, The University of Texas MD Anderson Cancer Center, Houston, TX, USA.

## Additional file

## Electronic supplementary material

Additional file 1: Table S1.: Multivariate analysis.(DOCX 33 KB)

Below are the links to the authors’ original submitted files for images.

Authors’ original file for figure 1

Authors’ original file for figure 2

Authors’ original file for figure 3

## Abbreviations

CTC: Circulating tumor cell
EMT: Epithelial-to-mesenchymal transition
EpCAM: Epithelial cell adhesion molecule
ER: Estrogen receptor
HER2: Human epidermal growth factor receptor 2
RECIST: Response Evaluation Criteria In Solid Tumors
SPSS: Statistical Package for Social Science
