# Supplementary material for: Circulating tumor cells as early predictors of metastatic spread in breast cancer patients with limited metastatic dissemination
Source: Breast Cancer Res. 2014 Sep 16;16:440. doi: 10.1186/s13058-014-0440-8 (PMC4303121; doi:10.1186/s13058-014-0440-8)
Supplement: Supplementary file 1 — Additional file 1: Table S1.: Multivariate analysis.(DOCX 33 KB) [file 13058_2014_440_MOESM1_ESM.docx]

**Additional file 1:Table S1. Multivariate analysis.**

| **Variable** | **Time to visceral disease^** | | **Time to new metastatic sites*** | | **Time to new metastatic lesions*** | |
| --- | --- | --- | --- | --- | --- | --- |
|  | **HR**  **(95% C.I.)** | **P** | **HR**  **(95% C.I.)** | **P** | **HR**  **(95% C.I.)** | **P** |
| **ER-positive *vs* negative** | 0.551  (0.340-0.893) | .016 | 0.375  (0.215-0.710) | .001 | 0.461  (0.286-0.742) | .001 |
| **HER2-positive *vs* normal** | 0.493  (0.270-0.900) | .021 | 0.365  (0.187-0.710) | .003 | 0.276  (0.149-0.512) | < .0001 |
| **Visceral metastases *vs* other** | - | - | 3.704  (1.862-7.368) | < .0001 | 3.435  (1.926-6.126) | < .0001 |
| **Bone metastases *vs* other** | 1.896  (1.128-3.189) | .016 | 2.282  (1.222-4.260) | .01 | 2.386  (1.396-4.078) | .001 |
| **Number of metastatic sites: 1 *vs* 2 *vs* ≥ 3** | 1.051  (0.756-1.462) | ns | **-** | **-** | **-** | **-** |
| **CTCs < 5 *vs* ≥ 5** | 0.596  (0.390-0.911) | .017 | 0.477  (0.293-0.775) | .003 | 0.416  (0.270-0.643) | < .0001 |

ER: estrogen receptor; HER2: human epidermal growth factor receptor 2; ns: statistically not significant

^ Time to visceral disease was evaluated in patients with disease originally confined to non-visceral organs

* Time to new metastatic sites and time to new metastatic lesions were evaluated in patients with disease originally confined to a single metastatic site.
