# Supplementary figures and images for: Circulating tumor cells as early predictors of metastatic spread in breast cancer patients with limited metastatic dissemination
Source: Breast Cancer Res. 2014 Sep 16;16:440. doi: 10.1186/s13058-014-0440-8 (PMC4303121; doi:10.1186/s13058-014-0440-8)

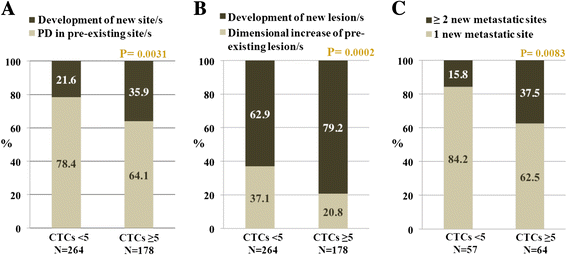

Supplement: Supplementary file 2 — Authors’ original file for figure 1 [file 13058_2014_440_MOESM2_ESM.gif]

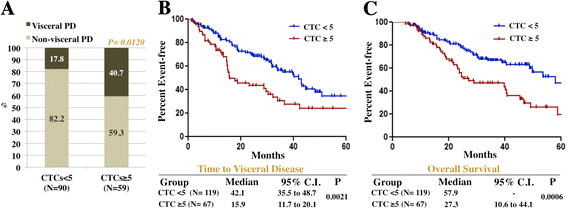

Supplement: Supplementary file 3 — Authors’ original file for figure 2 [file 13058_2014_440_MOESM3_ESM.gif]

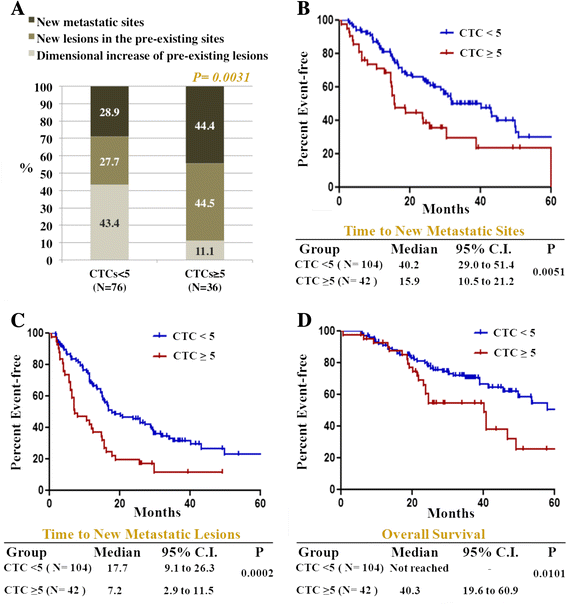

Supplement: Supplementary file 4 — Authors’ original file for figure 3 [file 13058_2014_440_MOESM4_ESM.gif]
